# First-Line Pazopanib Treatment in Metastatic Renal Cell Carcinoma: Real-World Data From a Single Chinese Center

**DOI:** 10.3389/fphar.2020.517672

**Published:** 2020-10-29

**Authors:** Bin Wang, Ji-Wen Song, Hui-Qing Chen

**Affiliations:** Department of Urology, Shanxi Cancer Hospital, Taiyuan, China

**Keywords:** carcinoma, renal cell, neoplasm metastasis, pazopanib, real-world, frist-line

## Abstract

The response to pazopanib in patients with metastatic renal cell carcinoma (mRCC) has been found to differ in Western and Eastern populations. Here, we analyzed the efficacy and side effects of pazopanib as first-line therapy in 31 consecutive patients with mRCC who were treated at a single Chinese center. Thirty-one consecutive patients with mRCC (20 males and 11 females, median age 59 years) were treated with pazopanib between October 2017 and July 2019. All patients had received a pathological diagnosis of RCC by prior radical nephrectomy or biopsy. All cases were treated with pazopanib (800 mg/day orally) as first-line therapy. Administration was continued until disease progression or unacceptable toxicities occurred. Objective response rate (ORR), disease control rate (DCR), progression-free survival (PFS), overall survival (OS), and safety were evaluated. Twenty-nine patients were eligible for final analysis. At the median follow-up of 12.7 months, 34.5% (10/29) patients achieved a partial response (PR), 41.4% (12/29) patients had stable disease (SD), seven (24.1%) patients had disease progression (PD), and one patient had died. The ORR and DCR were 34.5% and 75.9%, respectively, and the median PFS was 10.1 months (95% confidence interval, 4.1–17.7 months). OS could not be determined. The most common side effects were fatigue (11 cases, 37.9%), hand-foot syndrome (10 cases, 34.5%), change of hair color (10 cases, 34.5%), elevated alanine transaminase (ALT)/aspartate transaminase (AST) (10 cases, 34.5%), hypertension (seven cases, 24.1%), neutropenia (three cases, 10.3%), anemia (three cases, 10.3%), thrombocytopenia (two cases, 6.9%), and diarrhea (one cases, 3.4%). Major (grade 3 or higher) adverse events included hand-foot syndrome (two cases, 6.9%) and thrombocytopenia (one case, 3.4%). Most adverse events were ameliorated by dose reduction or treatment interruption. Remissions occurred in almost all patients with local recurrence or pulmonary metastases, whereas PD occurred in patients with bone, liver or brain metastases. Our real-world data suggest that pazopanib is definitely efficacious as first-line therapy for mRCC, with well-tolerated side effects. Different metastatic lesions may have different sensitivity to pazopanib. An additional, large sample, multicenter, prospective study is needed to confirm our results.

## Introduction

Renal cell carcinoma (RCC), which accounts for 2–3% of adult malignancies, has the highest mortality rate among all tumors of the urinary tract. Nearly one-third of patients with RCC have subjected to distant metastases at the time of the primary diagnosis and only 5% of patients with metastatic RCC (mRCC) having a 5-year overall survival rate. The treatment landscape of mRCC has evolved rapidly over the last decade, transitioning out of the cytokine era and into the era of tyrosine kinase inhibitor-targeted therapy. Traditional cytokine treatment has a low overall response rate and treatment cannot be sustained because of increasing serious adverse events. Compared with cytokine treatment, tyrosine kinase inhibitors have doubled the mean overall survival (OS) of patients with mRCC from 10 to 20 months and have become the standard of care for patients who are not candidates for resection (Randall et al., 2014). Pazopanib, a multi-targeted tyrosine kinase inhibitor with both anti-angiogenic and anti-tumor properties, is a standard first-line drug for RCC and can achieve up to 30% objective response in advanced RCC patients (Cella and Beaumont, 2016). From October 2017 to July 2019, pazopanib was used as first-line therapy in 31 patients with mRCC in our hospital, and efficacy, safety and effectiveness against different metastatic lesions were analyzed.

## Materials and Methods

In this retrospective study, 31 consecutive patients with mRCC were treated with pazopanib between October 2017 and July 2019. Each patient had received a pathological diagnosis of RCC, with prior radical nephrectomy or biopsy. Patients treated with pazopanib for less than two cycles were excluded from the study. Objective response rate (ORR), disease control rate (DCR), progression-free survival (PFS), OS, and safety were evaluated in this study. Ethical approval for the study was given by the Institutional Review Board and Ethics Committee of Shanxi Cancer Hospital. However, since this is a retrospective study, no human specimens were involved and data acquisition was carried out anonymously.

### Clinical Information

A total of 31 consecutive patients (20 males and 11 females), between 32 and 77 years old (median age 59), were enrolled in the study. Twenty-five patients had undergone a primary tumor resection and another six had undergone a color Doppler ultrasound-guided tumor biopsy. The pathological diagnosis in each case was confirmed as RCC. Most of the patients (22 patients, 71%) had an Eastern Cooperative Oncology Group (ECOG) performance status score of 0 or 1. Ten patients had favorable-risk, 16 patients had intermediate-risk, and five patients had poor-risk disease, assessed by the Memorial Sloan-Kettering Cancer Center (MSKCC) criteria. The median number of metastatic sites was 1 (range, 1–5). Metastatic lesions included lung metastases, bone metastases, liver metastases, brain metastases, local recurrence, adrenal metastases, and subcutaneous metastases. The characteristics of patients are presented in Table 1.

**TABLE 1:** Baseline patient characteristics (*n* = 31).

Variables	Number of patient (%)
Median age (range)	59 (32–77)
Sex	
Male	20 (64.5)
Female	11 (35.5)
Histology	
Clear cell carcinoma	27 (87.1)
Non-clear cell carcinoma	4 (12.9)
Comorbidities	
Hypertension	6 (19.4)
Diabetes	7 (22.6)
Others	2 (6.5)
Previous nephrectomy	
Yes	25 (80.6)
No	6 (19.4)
ECOG performance status	
0	3 (9.7)
1	19 (61.3)
2	7 (22.6)
3	2 (6.5)
MSKCC risk group	
Favorable	10 (32.3)
Intermediate	16 (51.6)
Poor	5 (16.1)
Number of metastasis	
1	21 (67.7)
≥2	10 (32.3)
Site of metastasis	
Lung	16 (51.6)
Bone	8 (25.8)
Liver	2 (6.5)
Brain	4 (12.9)
Local recurrence	7 (22.6)
Adrenal	3 (9.7)
Subcutaneous	3 (9.7)

### Therapeutic Protocol

All patients received pazopanib (800 mg/day) orally. Three patients had the dose reduced because of adverse events of grade 3 or above. During treatment, the dose of pazopanib was adjusted or suspended, according to the severity of intolerable side effects.

### Evaluation of Efficacy and Adverse Events

Based on the Response Evaluation Criteria in Solid Tumors (RECIST) 1.1, efficacy was evaluated every 8 weeks and categorized as complete response (CR), partial response (PR), stable disease (SD), or disease progression (PD). The beneficial effects of the treatment were required to be sustained for at least 4 weeks. The primary end points were ORR, DCR, and PFS and the secondary end points were OS and adverse events. The criteria for evaluation of drug toxicity were those set out in the Common Terminology Criteria for Adverse Events (CTCAE) version 4.03, and safety assessments were carried out every 4 weeks. Prior to treatment, medical history, physical examination and the baseline images of the target lesion were recorded in detail. Complete blood counts and serum chemistry tests were carried out weekly and thyroid function was measured every 4 weeks while receiving pazopanib. Vital signs, body weight, and ECOG scores were monitored, and the severity of adverse drug reactions, and the time of their occurrence or disappearance, were recorded. Imaging studies were performed every 8 weeks and computed tomography (CT) scans were used as the main evaluation tool for measuring the maximum change in diameter of measurable lesions.

### Statistical Methods

Data processing was performed using SPSS 17.0 software. PFS and OS were analyzed using Kaplan-Meier methods. The significance level was set at alpha = 0.05.

## Results

### Objective Efficacy

All patients were followed up for a median of 12.7 months (range, 3–22 months). At the time of analysis, 29 patients were eligible for the final therapeutic evaluation since they have completed more than 8 weeks’ treatment. Evaluation of efficacy showed that 34.5% (10/29) patients achieved PR, 41.4% (12/29) patients maintained SD, and 24.1% (7/29) patients had PD. None of these patients achieved CR and one patient died. Overall, the ORR was 34.5% and the DCR was 75.9%. During follow-up, 16 patients experienced PD. One patient died of later stage cancer 6.0 months after treatment. The median time to progression was 10.1 months (95% confidence interval, 4.1–17.7 months). The OS had not been reached.

### Side Effects

The most common side effects were fatigue (11 patients, 37.9%), hand-foot syndrome (10 patients, 34.5%), change of hair color (10 patients, 34.5%), elevated alanine transaminase (ALT)/aspartate transaminase (AST) (10 patients, 34.5%), hypertension (seven patients, 24.1%), neutropenia (three patients, 10.3%), anemia (three patients, 10.3%), thrombocytopenia (two patients, 6.9%), and diarrhea (one patient, 3.4%). Most adverse events were grade 1–2; grade 3 or higher adverse events included two cases of hand-foot syndrome (6.9%) and one case of thrombocytopenia (3.4%). Fatigue, hand-foot syndrome, change of hair color, and elevated ALT/AST were the most common adverse events (Table 2).

**TABLE 2:** Adverse events due to pazopanib.

Toxicity	All grades (%)	Grade ≥3
Fatigue	11 (37.9%)	
Hand-foot syndrome	10 (34.5%)	2 (6.9%)
Hair color change	10 (34.5%)	
Raised ALT/AST	10 (34.5%)	
Hypertension	7 (24.1%)	
Neutropenia	3 (10.3%)	
Anemia	3 (10.3%)	
Thrombocytopenia	2 (6.9%)	1 (3.4%)
Diarrhea	1 (3.4%)	

### Dose Reduction and Treatment Interruption

Eight patients (27.6%) had their medication reduced or withdrawn over the course of the treatment, although treatment was not suspended for more than 2 weeks. Adverse events in all patients could be controlled and made tolerable by symptomatic support, dose reduction, or drug withdrawal. Because of grade 3 or higher adverse events, three (10.3%) patients had their dose of pazopanib reduced and completed the treatment program on a dose of 600 mg/day.

### Clinical Efficacy Against Different Metastatic Lesions

Remissions were achieved in the majority of patients with local recurrence (40%) or pulmonary (60%) metastases. PD occurred mainly in patients with bone (28.6%), liver (28.6%), and brain (42.9%) metastases. Among 10 patients with PR, six cases with pulmonary metastases and four cases with local recurrence entered remission. One of the patients with PD (the case with liver and brain metastases) was treated for 4 months. Imaging studies then showed an increase in metastatic tumor and the patient died of tumor exhaustion at 6 months.

## Discussion

RCC is a highly vascularized malignant tumor, the pathogenesis of which involves inactivation of the *VHL* gene, which promotes high expression of the most important vascular endothelial growth factor (VEGF), platelet-derived growth factor (PDGF), transforming growth factor, and other proteins associated with neovascularization. All of these factors play critical roles in the development and metastasis of tumors (Lang and Harrison, 2010). With further understanding of the biological and molecular pathogenesis of RCC, a variety of molecular targeted medicines have recently been introduced and have produced remarkable outcomes in the treatment of mRCC. Pazopanib is indicated in adult patients with mRCC, both as a first-line treatment and after previous treatment with cytokines (Park et al., 2018). Several clinical trials have provided evidence of positive responses to pazopanib as first-line therapy for mRCC. Treatment with pazopanib gave 8 months PFS compared with placebo and 11 months PFS compared with sunitinib (Cecerev et al., 2016; Lalani et al., 2017). The COMPARZ study demonstrated the non-inferiority of pazopanib compared with sunitinib in terms of PFS and OS. This evidence is consistent with that obtained in a retrospective study and also in the real-life retrospective SPAZO study, which showed an ORR of 30.3% and PFS of 11.1 months (Ruiz-Morales et al., 2016). The PISCES study showed that toxicity can influence both patient and clinician preference for pazopanib over sunitinib (Méndez-Vidal et al., 2018). Reports from a number of retrospective observational studies, carried out in Europe, on the outcomes of mRCC patients treated with pazopanib have been published in the last few years. The effects of pazopanib have also been studied in Asian subpopulations. Joshi et al. reported that 28 consecutive patients with mRCC, who were treated with first-line pazopanib in India, achieved an overall clinical benefit rate (PR + SD) of 76% and a median PFS of 5.9 months (Joshi et al., 2016). Rudresha et al. (2017) reported that, in a single institutional review of 40 mRCC patients treated with three different tyrosine kinase inhibitors, sunitinib, pazopanib and sorafenib, ORR, median PFS and OS of pazopanib-treated patients were 36.3% and 11.2 and 20.1 months, respectively. A larger, multi-center, real-world, retrospective study carried out in Korea by Kim et al. (2016) found similar ORR (59%), median PFS (12.2 months), and OS (21.9 months). Another Korean multi-center study carried out by Kim et al. (2018) also demonstrated that pazopanib improves ORR (36.4%) and prolongs PFS (10.1 months) and OS (40.2 months). To the best of our knowledge, there are, to date, very few published reports describing pazopanib treatment of Chinese patients with mRCC. Real-world data for contemporary treatment experiences using pazopanib, including adverse events, in an unselected patient setting, have been lacking.

In contrast to a clinical trial, our study involved an unselected cohort of patients. In this group, the ORR of pazopanib as first-line treatment for mRCC was 34.5% and the median PFS was 10.1 months, which are similar to the findings of other studies. Ethnic and genetic difference may, however, lead to subtle differences in ORR, PFS, and OS. Patients in our study who benefitted from treatment were mostly suffering from local recurrence and pulmonary metastases, whereas patients whose disease progressed mainly had bone, liver, and brain metastases, suggesting that there may be some differences in the efficacy of pazopanib against metastases in different organs and tissues.

The rapid proliferation of tumor cells leads to a state of hypoxia, or lack of oxygen. Delivery of oxygen and continued proliferation of the tumor can be achieved by angiogenesis, promoted by downstream products of hypoxia-inducible factor (HIF), such as VEGF, PDGF, and EPO. Solid tumors are extremely sensitive to hypoxia and the growth of pulmonary metastases, soft tissue metastases and other solid lesions, which are especially dependent on angiogenesis, may be more dependent on the VHL-HIF-hypoxia response gene pathway (Matrana et al., 2016). Pazopanib can arrest the migration and proliferation of vascular endothelial cells and tumor angiogenesis by blocking VEGF, PDGF, and other molecular targets. Pazopanib is, thus, effective against pulmonary and soft tissue metastases and other solid metastatic lesions.

The inhibition of many targets by pazopanib has the potential to cause numerous adverse events. Hematologic toxicity and hepatic toxicity, in particular, are serious adverse events and should be paid attention (Jeon et al., 2019). There is, however, still no definitive evidence to support a relationship between severity of adverse events and efficacy. The toxicity profile in our study group suggests better tolerance and safety in our patients than in patients in other trials. Only 27.6% of our patients required dose reduction, compared with 44% patients in the COMPARZ study. The most significant toxicity of pazopanib in our study population was hand-foot syndrome, which was the reason for dose reduction in most patients. The incidence of hand-foot syndrome (34.5% vs. 29%), change in hair color (34.5% vs. 30%), and elevated ALT/AST (34.5% vs. 60%) in our study was similar to that in the COMPARZ study, while all other toxicities were encountered much less frequently in our study (Guo et al., 2018). Only one of our patients, who had grade 3 or 4 thrombocytopenia and required blood transfusion, showed severe hematological toxicity. Most adverse events were transient, and there were few severe non-hematological toxicities, with grade 3 or 4 hand-foot syndrome occurring in only 6.9% of patients.

### Limitations

There are some limitations to this study. Firstly, it was a retrospective study at a single cancer center in China, so our results may not be generalized to other institutions or locations. Secondly, the sample size is relatively small and long-term follow-up data such as OS are lacking. An additional large sample, multicenter, prospective study is, therefore, needed to confirm our results.

## Conclusion

The present study further confirms that pazopanib is efficacious as a first-line treatment for patients with mRCC and has controllable and reversible side effects. We also found that metastatic lesions in different organs and tissues may have different sensitivity to pazopanib. An increased sample size and extended follow-up period are needed to further observe the survival benefits conferred by pazopanib.

## Data Availability Statement

The original contributions presented in the study are included in the article/supplementary material, further inquiries can be directed to the corresponding author/s.

## Author Contributions

Study concepts: BW, JS, and HC. Study design: BW, JS, and HC. Data acquisition: BW, JS, and HC. Data analysis and interpretation: BW. Statistical analysis: BW. Manuscript preparation: BW, JS, and HC. Manuscript editing: BW. Manuscript review: JS and HC. All authors read and approved the final manuscript.

## Conflict of Interest

The authors declare that the research was conducted in the absence of any commercial or financial relationships that could be construed as a potential conflict of interest.
